# Biphenotypic human papillomavirus-associated head and neck squamous cell carcinoma: a report of two cases

**DOI:** 10.1186/s13000-015-0334-9

**Published:** 2015-07-11

**Authors:** Gayani Pitiyage, Mary Lei, Teresa Guererro Urbano, Edward Odell, Selvam Thavaraj

**Affiliations:** Head and Neck Pathology, 4th Floor Tower Wing, Guy’s and St. Thomas’ NHS Foundation Trust, Great Maze Pond, London, SE1 9RT UK; Department of Clinical Oncology, Lower Ground Floor, Lambeth Wing, Guy’s and St Thomas’ NHS Foundation Trust, St Thomas’ Hospital, Westminster Bridge Rd, London, SE1 7EH UK; Mucosal and Salivary Biology, King’s College London Dental Institute, 4th Floor Tower Wing, Great Maze Pond, London, SE1 9RT UK

**Keywords:** Biphasic, Biphenotypic, Differentiated, Human papillomavirus, Oropharyngeal, Squamous cell carcinoma, Undifferentiated, Variant

## Abstract

Human papillomavirus-associated oropharyngeal squamous cell carcinoma is now recognised as a subtype of head and neck cancer with distinct clinical, molecular and histological characteristics. The majority of these carcinomas are of non-keratinising squamous type but there is a growing number of histomorphologic variants of this disease. Here we describe the clinical, histomorphologic and immunophenotypic features of two cases of human papillomavirus-associated oropharyngeal squamous cell carcinoma demonstrating a clearly delineated biphasic differentiated and undifferentiated phenotype.

## Background

Oropharyngeal squamous cell carcinoma (OpSCC) associated with high-risk subtypes of human papillomavirus (HPV) demonstrates distinct demographic and clinical characteristics. These carcinomas often arise in non-smokers who do not consume alcohol to excess and present on average 5–6 years earlier than site and staged matched HPV-negative carcinomas [1]. Importantly, patients with HPV-associated OpSCC have significantly improved overall and disease-specific survival compared to HPV-negative controls. Alongside these observations, HPV-associated OpSCC have also been shown to contain fewer cumulative mutations and demonstrate particular histomorphological features [2, 3], recapitulating the reticulated crypt of Waldeyer’s ring and lacking keratin with little or absent squamous maturation [4]. Other microscopic features strongly predictive of HPV-association are an architectural arrangement as broad interconnecting strands, large islands or sheets, a well-delineated invasive front and lack of significant stromal desmoplasia [5]. These clinicopathological features have led some authorities to call for recognition of HPV-associated OpSCC as a distinct entity in future classifications [6].

While the majority of HPV-associated OpSCC are of non-keratinising type, a growing number of divergent morphological types have been described recently. These include basaloid squamous cell carcinoma, papillary squamous cell carcinoma, adenosquamous carcinoma, adenocarcinoma, lymphoepithelial carcinoma and small cell neuroendocrine carcinoma [7–12]. These reports are limited to isolated cases or small series and the clinical and biological significance of these subtypes are yet to be elucidated, particularly whether these histological subtypes have similar prognostic implications to conventional non-keratinising OpSCC. Defining the morphological diversity of HPV-associated OpSCC is necessary to determine the clinical behaviour of these possible variants.

We describe the clinical, histomorphologic and immunophenotypic features of two cases of HPV-associated OpSCC demonstrating a clearly delineated biphasic differentiated and undifferentiated phenotype.

## Case presentation

### Case 1

A fifty-nine year old male presented with a 4 × 2 cm lump in the right neck at level II. He had noticed the swelling for less than one week but reported a three year history of sore throat, fatigue, reduced exercise tolerance and shortness of breath. He had never smoked and consumed 18 international units of alcohol per week. There was no other lymphadenopathy and no intra-oral abnormality. Fine needle aspiration cytology was inconclusive and the patient underwent a diagnostic lymph node excision for histological evaluation, which showed metastatic squamous cell carcinoma. Computed tomography (CT) and ^15^fluoro-deoxyglucose positron emission tomography-computed tomography (FDG-PET/CT) were undertaken to identify the primary site and revealed a 2 cm mass involving the right tonsil with a right sided level II lymph node mass extending into the deep lobe of the right parotid gland. He was treated with radical chemoradiation comprising image-guided intensity modulated radiotherapy (IMRT) delivering 65Gy in 30 fractions to the oropharynx and right neck level II with 54Gy in 30 fractions to right neck levels III to V and left neck levels II to V. Concomitant cisplatin 100 mg/m^2^ was given on days 1 and 29 of radiotherapy. A FDG-PET/CT scan performed at 3 months post-treatment confirmed a complete metabolic response. The patient remains disease free after 15 months.

#### Histology and immunohistochemistry

**Fig. 1:**
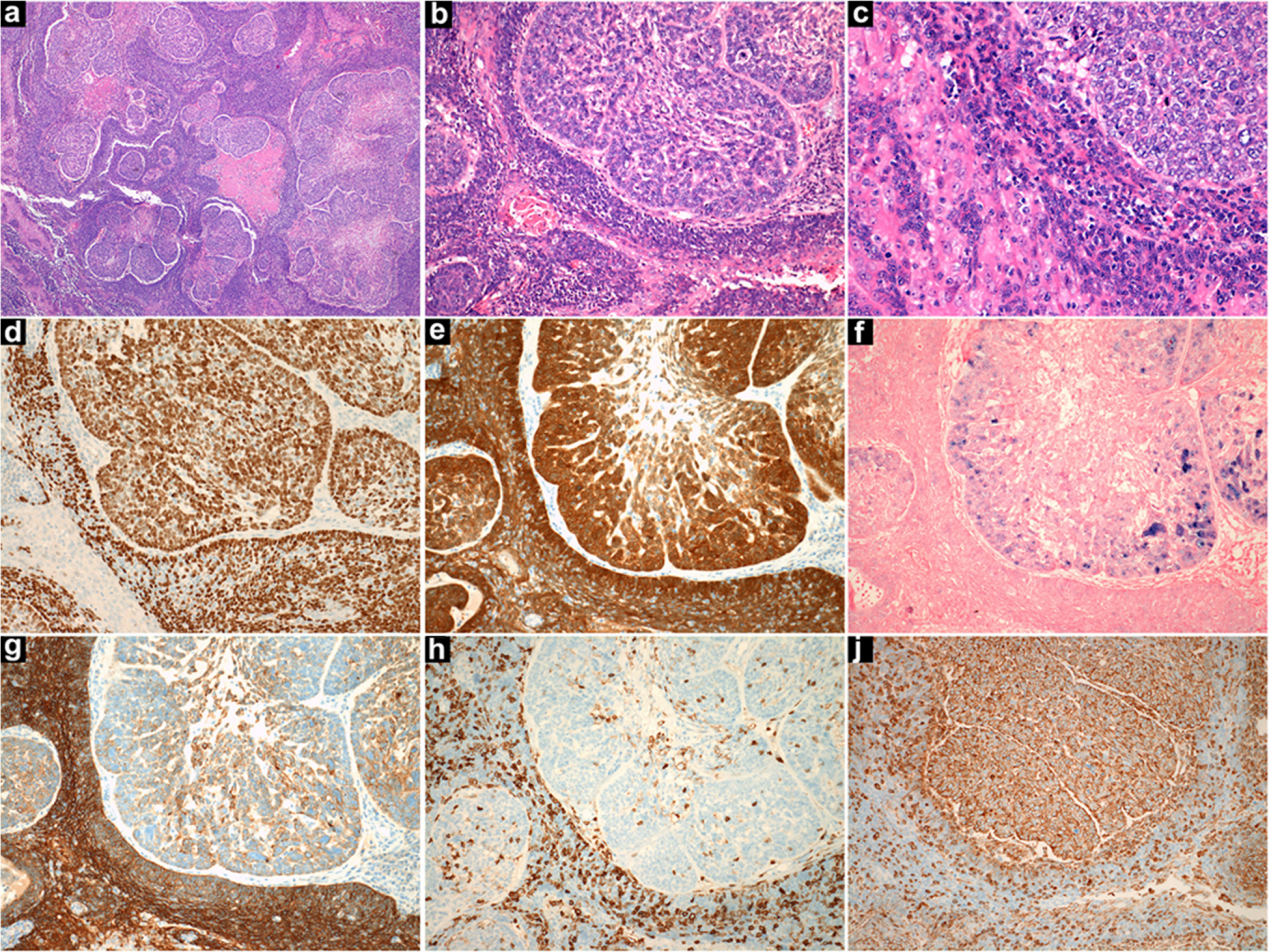
Representative histological and immunohistochemical photomicrographs of Case 1. **a-c**. Low, medium and high power views (original magnification x25, x100 and x200, respectively, H&E), **d**. p63, **e**. p16, **f**. high-risk HPV ISH, **g**. CK5/6, **h**. CD45, **j**. Vimentin

Histological evaluation of the excised lymph showed complete effacement by carcinoma comprising two clearly demarcated and morphologically distinct subpopulations of cells. In most areas, the two subpopulations were separated by fibrous tissue bands. Where the two phenotypes were not separated by fibrous tissue, there was abrupt delineation of the subpopulations without any intermediate transition. One component was composed of broad interconnecting strands of tumour cells with obvious squamous maturation but with minimal keratinisation typical of conventional HPV-associated OpSCC, designated the ‘differentiated’ component. The second component, referred to as the ‘undifferentiated’ component, was arranged as syncytial sheets of cells with comparatively large vesicular nuclei (Fig. 1a, b and c). Immunohistochemically, both components demonstrated strong nuclear positivity for p63 (1:50, Clone 4A4, Santa Cruz) and strong nuclear and cytoplasmic staining for p16 (pre-diluted, CINTec, MTM Laboratories) in greater than 70 % of tumour cells (Fig. 1d and e). While the differentiated component demonstrated strong and moderate staining for CK5/6 (1:25, Clone D5/16 B4, Dako; Fig. 1g) and Vimentin (1:500, Clone V9, Dako; Fig. 1j), respectively, expression of these proteins were weak to absent in the undifferentiated component. Similarly, while there was moderate protein expression of CK8/18 (1:2, Clone Cam5.2, Dako) and CK19 (1:50, Clone RCK108, Dako) in the differentiated areas, there was no staining for these cytokeratins in the undifferentiated component (data not shown). Both components were negative for CK7 (1:200, Clone OV-TL 12/30, Dako) and CK20 (1:25, Clone K_s_ 20.8, Dako; data not shown). A modified quick score was used for semi-quantification of immunohistochemical staining. Briefly, the proportion and intensity of staining was quantified from 0–5 and 0–3, respectively. The quick score is the sum of the proportion and intensity giving a range of 0–8 [13]. There was also a greater proliferation index (Ki-67; 1:50, Clone MIB-1, Dako; data not shown) and a denser population of tumour infiltrating lymphocytes (TILs; CD45, 1:100, Dako; Fig. 1h) in the differentiated component in this case. By contrast, the undifferentiated cells showed strong diffuse nuclear staining for high-risk HPV DNA (Inform HPV III Family 16 probe, Ventana Medical Systems) suggestive of episomal viral DNA while the differentiated cells only showed punctate nuclear staining in a pattern consistent with viral integration (Fig. 1f).

### Case 2

A fifty-four year old male presented with an eight month history of right-sided level II neck lump. He had a life-long history of smoking 10 cigarettes a day and heavy alcohol use of 40 international units per week. Examination revealed a 2 cm right sided tonsillar mass extending to the tonsillar pillar and the soft palate with a 6 × 4 cm right sided neck mass spanning levels II and III. Tonsil biopsy confirmed squamous cell carcinoma. The patient was treated with two 3-weekly cycles of induction chemotherapy comprising cisplatin 80 mg/m^2^ on day 1 and continuous infusional 5-fluorouracil 1000 mg/m^2^ on days 1–4, followed by radical chemoradiation. IMRT consisted of 65Gy in 30 fractions to the oropharynx and right neck levels Ib, II and the upper part of level III and 54Gy in 30 fractions to the right neck lower level III, IV and V and left neck levels II to V. Concomitant cisplatin 100 mg/m^2^ was delivered on days 1 and 29 of radiotherapy. A 3 month post-treatment FDG-PET-CT scan showed an equivocal FDG uptake of 4.5 maximum Standardised Uptake Variable (SUV) noted at the original site but with no mass lesion correlated on CT imaging and only low-grade FDG uptake in minimally enlarged right sided level II neck nodes. A multidisciplinary review recommended surveillance with repeat FDG-PET/CT scanning after three months.

#### Histology and immunohistochemistry

**Fig. 2:**
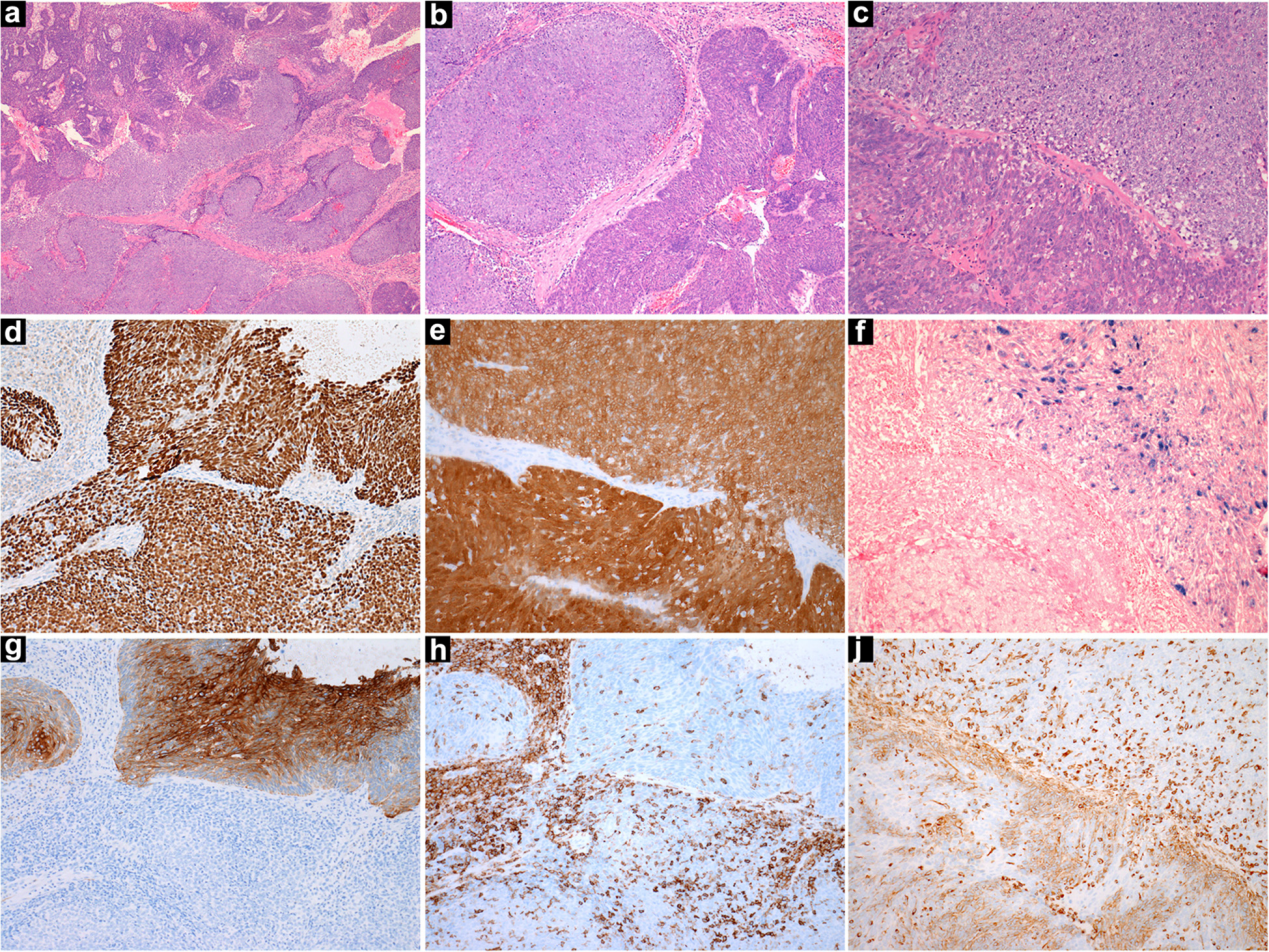
Representative histological and immunohistochemical photomicrographs of Case 2. **a-c**. Low, medium and high power views (original magnification x25, x100 and x200, respectively, H&E), **d**. p63, **e**. p16, **f**. high-risk HPV ISH, **g**. CK7, **h**. CD45, **j**. Vimentin

Incisional biopsy of the right tonsil showed a biphenotypic carcinoma with features similar to those of Case 1. In addition, the differentiated component demonstrated a focal papillary architecture lacking keratinisation and centred on the tonsillar crypts (Fig. 2a, b and c). Both components demonstrated strong nuclear positivity for p63 and strong nuclear and cytoplasmic staining for p16 in greater than 70 % of tumour cells (Fig. 2d and e). By contrast to Case 1, the differentiated component showed greater percentage and intensity of staining for CK7 (Fig. 2g) and CK19. Furthermore, unlike Case 1, both components showed similar percentage and intensity for CK8/18 and similar percentage of cells in cycle (data not shown). Also by contrast to Case 1, high-risk HPV ISH showed diffuse nuclear staining in the differentiated compartment and a punctate pattern in the undifferentated areas (Fig. 2f). A slightly lower density of TILs was present in the former component (Fig. 2h). The relative expression of Vimentin in the two components was similar to that of Case 1 (Fig. 2j). The immunohistochemical profiles for cases 1 and 2 are summarised in Table 1.

**Table 1:** Summary of patient demographic, clinical, follow-up data as well as immunohistochemical and in-situ hybridisation profiles. The immunohistochemical profile is summarised as follows: quick score 0 = −, 1–2 = +, 3–5 = ++, 6-8 = +++. *Strong diffuse nuclear and cytoplasmic staining in >70 % of tumour cells

	Case 1		Case 2	
Age	59		54	
Sex	Male		Male	
TNM	T2 N2b		T1 N2b	
Treatment	Concomitant chemo-radiotherapy		Concomitant chemo-radiotherapy	
Follow-up	18 months		6 months	
Immunophenotype	Differentiated component	Undifferentiated component	Differentiated component	Undifferentiated component
AE1/3	+++	+++	+++	+++
CK5/6	+++	+	+++	+++
CK7	-	-	++	-
CK8/18	+++	+	++	++
CK19	+++	-	+++	++
CK20	-	++	-	-
Vimentin	+++	++	++	++
Ki-67	+++	++	+++	+++
p63	+++	+++	+++	+++
p16*	+++	+++	+++	+++
HR-HPV ISH	Fine, punctate	Strong, diffuse	Strong, diffuse	Fine, punctate
TILs	+++ (>75 %)	+ (25-50 %)	+ (25-50 %)	++ (50-75 %)

## Discussion

Determining the HPV status of OpSCCs has important prognostic significance since it is now well established that patients with HPV-associated tumours have improved outcome compared to site-matched HPV-negative controls [14, 15]. In addition to distinctive clinical and demographic features, HPV-associated OpSCC has particular histological features. They recapitulate the tonsil reticulated crypt epithelium, lack keratinisation, form syncytial sheets with inconspicuous intercellular bridges and often contain areas of central necrosis. Furthermore, these tumours invade as sheets, broad interconnecting strands or large islands, lack stromal desmoplasia and have a rich tumour-infiltrating lymphocyte (TIL) population. [5, 6]. Chernock et al. used the term ‘non-keratinising’ to describe these features and demonstrated a strong positive correlation with HPV status [2]. Interestingly, this non-keratinising morphology predicts improved survival and has strong inter-observer reliability among pathologists [2, 16]. In a subsequent review article, Chernock postulated that recognition of these histological features may be of benefit in clinical situations where p16 immunohistochemistry or HPV-specific testing are not available [5]. However, it is important to note that using histomorphological criteria to predict HPV status, and thereby as a prognostic indicator’ is limited to the ‘keratinising’ and ‘non-keratinising’ subtypes of OpSCC.

While the great majority of HPV-associated OpSCCs are of ‘non-keratininsing’ type, there is a growing number of reports detailing divergent phenotypes including basaloid squamous cell carcinoma (SCC), papillary SCC, adenosquamous carcinoma, adenocarcinoma, undifferentiated (lymphoepithelial) carcinoma and small cell neuroendocrine carcinoma [7–12, 17–20]. However, further work is necessary to determine whether the biphenotypic variant has prognostic implications compared with conventional HPV-associated OpSCC.

There have been recent calls to replace the current World Health Organisation OpSCC grading system of well-, moderately- and poorly-differentiated with a three tier system of ‘keratinising SCC’, ‘non-keratinising SCC’ and ‘non-keratinising SCC with maturation’ [5, 6, 16, 21]. The latter, which has also been termed ‘hybrid SCC’, is described as ‘consisting of definitive areas with non-keratinising SCC morphology but also having maturing squamous differentiation comprising >10 % of tumour surface area.’ [16]. In this subtype, there is a gradual transition from conventional areas of non-keratinising SCC to area with squamous maturation or ‘frank keratinisation’. There are several differences between the cases described in the current report and the ‘non-keratinising SCC with maturation’. In the former, two phenotypes are morphologically and immunophenotypically distinct and there is lack of transition between the differentiated and undifferentiated components; a zone of fibrous tissue almost always separates the two areas (Figs. 1a, b, c, 2a, b and c). Furthermore, in the current cases the ‘undifferentiated’ component demonstrated features similar to those described as HPV-associated lymphoepithelial carcinoma [11]. These features do not fulfil the criteria of a ‘non-keratinising SCC with maturation’ as originally described and the cases in the current report are histomorphologically distinct [5, 16].

In the two cases reported here, both components demonstrated similar staining patterns for p16, p63, CK19 and Vimentin. Interestingly, in Case 1, strong staining for high-risk HPV DNA ISH, a function of viral copy number [22], was present in the undifferentiated component and co-localised with weak CK5/6 staining, lack of CK7 expression and a low TIL density (Fig. 1f, h and Table 1). By contrast, Case 2 showed strong ISH staining in the differentiated component and was associated with strong CK5/6, moderate CK7 expression and a moderate density of TILs (Fig. 2f, h and Table 1). This suggests that the two phenotypes are not explained by HPV physical status or copy number alone. Recent reports demonstrate the prognostic utility of TILs in HPV-associated OpSCC [23–25]. The current cases pose a prognostic dilemma since the TIL density varied markedly between component phenotypes. The lack of consistent correlation between morphological and immunophenotype with viral copy number in the current two cases raises the possibility that factors other than relative HPV oncoprotein expression are likely to influence the biological behaviour of this disease.

## Conclusion

We describe two cases of biphasic HPV-associated OpSCC demonstrating distinct differentiated and undifferentiated histological phenotypes. To our knowledge, this is the first report of this entity in the literature and adds to the growing number of histological variants of HPV-associated OpSCC. Further work is required to determine the prognostic significance of this variant.

## Consent

Written informed consent was obtained from the patients for publication of this case series and accompanying images. A copy of the written consent is available for review by the Editor-in-Chief of this journal.

## Abbreviations

CK: Cytokeratin
FDG-PET/CT: ^15^fluoro-deoxyglucose positron emission tomography-computed tomography
HPV: Human papillomavirus
OpSCC: Oropharyngeal squamous cell carcinoma
TIL: Tumour-infiltrating lymphocytes
